# Antioxidant and Antimicrobial Activity of 5-methyl-2-(5-methyl-1,3-diphenyl-1*H*-pyrazole-4-carbonyl)-2,4-dihydro-pyrazol-3-one

**Published:** 2009-12

**Authors:** K. B. Umesha, K. M. L. Rai, M. A. Harish Nayaka

**Affiliations:** 1 *Department of Chemistry, Yuvaraja’s College, University of Mysore, Mysore, India;*; 2 *Department of Studies in Chemistry, University of Mysore, Manasagangotri, Mysore, India;*; 3 *Department of Studies in Sugar Technolgy, Sir M. V. Post Graduate Center, University of Mysore, Mandya, Karnataka, India*

**Keywords:** pyrazoles, antimicrobial, antioxidant

## Abstract

Cycloaddition of nitrile imines 4 generated *in situ* by the catalytic dehydrogenation of diphenyl hydrazones 3 using Chloramine-T (CAT) as oxidant in glacial acetic acid with enolic form of ethyl acetoacetate 5 afforded Ethyl 3-aryl-5-methyl-1-phenyl-1*H*-pyrazol-4-carboxylate 6 in 80% yield. The said pyrazoles 6 refluxed with 80% hydrazine hydrate using absolute alcohol as solvent for about 2–3 hours to produce the respective 5-methyl-1,3-diphenyl-1*H*-pyrazole-4-carboxylic acid hydrazide 7. The alcoholic solution of pyrazole acid hydrazides on heating with ethyl acetoacetate 5 to give the 5-methyl-2-(5-methyl-1,3-diphenyl-1*H*-pyrazole-4-carbonyl)-2,4-dihydro-pyrazol-3-one 8. The synthesized compounds were found to exhibit good antimicrobial and antioxidant activity as evaluated by 1,1-diphenyl-2-picryl Hydrazyl (DPPH) radical scavenging, reducing power and DNA protection assays.

## INTRODUCTION

Heterocyclic compounds are considered as the most promising molecules for the design of new drugs. 1,3-dipolar cycloaddition reaction is an efficient synthetic tool for constructing biologically potent five membered heterocyclic compounds (1, 2). Pyrazole derivatives are associated with wide spectrum of biological activities such as analgesic, antipyretic, anti-inflammatory, antifungal, antimicrobial, antiprotozoal, antiviral, antitubercular, anticoagulant, CNS-depressant, anti-HIV, anticancer, COX-2-inhibitors [Celecoxib, Rofecoxib, Etoricoxib], herbicidal and plant growth regulating (3–8) properties. Apart from the various dipolar reagent known, nitrile imines are used in numerous 1,3-dipolar cycloaddition reaction leading to pyrazoles, pyrazolines, pyrazolidines and other heterocyclic compounds (9).

Huisgen and co-workers first reported (10) the authentic *in situ* generation of nitrile imines by the thermolysis of 2,5-diphenyl tetrazole in the presence of ethyl phenyl propiolate and obtained 2,3,5-triphenyl carbethoxypyrazole. The usual synthesis of nitrile imines involves the thermolysis or photolysis of tetrazole (10), oxidation of aldehyde hydrazones with lead tetra acetate (11), CAT (12) and mercuric acetate (13). Photolysis of 3,4-disubstituted sydnones and 2,4-disubstituted-1,3,4-oxadiazolin-5-ones (14) also provide nitrile imines.

In addition to this, nitrile imines are known to react with heterocyclic compounds to yield a variety of polyheterocycles (14). Shawali and Co-workers (15) prepared a numerous pyrazole derivatives by the reaction of *in situ* generated nitrile imines obtained from hydrazidoyl halides with sodium salt of active methylene compounds, such as *β*-ketosulphones, *β*-ketoanilides and *β*-cyano ketones. Baruah *et al* (16) generated the *C*-acetyl and *C*-ethoxycarbonyl nitrile imines *in situ* from the corresponding hydrazonoyl halides in the presence of dry triethylamine in anhydrous chloroform, and have used these nitrile imines for the preparation of pyrazoles derivatives. The intramolecular cycloaddition of *in situ* generated nitrile imine with aldonitrones afforded triazoles (17). Mogilaiah *et al* (18) developed a solvent free method for the facile synthesis of 1,8-naphthyridinyl-pyrazoles using POCl_3_-DMF (Vilsmeier-Haack reagent) over silica gel under microwave irradiation. Aly *et al* (19) showed a new synthetic route for the synthesis of some noval pyrazole derivatives from 3-aryl-1-phenyl-*1H*-pyrazole-4-carbaldehydes. Desai and co-workers (20) synthesized the antibacterial and antifungal activities of some 4-{1-aza-2[(aryl)]amino}-3-methyl-1-(phenyl)-2-pyrazolin-5-ones from ethyl(2E)-2-acetyl-3-aza-3-[(4-bromophenylamino)]prop-2-enolate on treatment with phenyl hydrazine.

Padmavathi and Co-workers (21) prepared activated bis pyrazolines and bis isoxazolines by 1,3-dipolar cycloaddition of nitrile imines and nitrile oxides to activated bis olefinic systems in the presence of Chloramine-T. Bacchetti (22) prepared 1,4-dicarboethoxy pyrazoles by intermolecular cycloaddition of nitrile imines with ethyl acetoacetate. Though there are more references available in the literature on cycloaddition of nitrile imines with alkenes and alkyne, there is a less information about the use of keto-enol tautomers as dienophile for the cycloaddition. we have synthesized (23) the 4-acetyl-5-methyl-1,3-diphenylpyrazoles in quantitative yield via 1,3-dipolar cycloaddition of enol form of acetyl acetone with the nitrile imines generated *in situ* by the catalytic dehydrogenation of diphenyl hydrazones using CAT. In view of these reports, we synthesized 5-methyl-2-(5-methyl-1,3-diphenyl-1*H*-pyrazole-4-carbonyl)-2,4-dihydro-pyrazol-3-one (8) and evaluated for antioxidant and antimicrobial activity.

## MATERIALS AND METHODS

**Figure 1:**
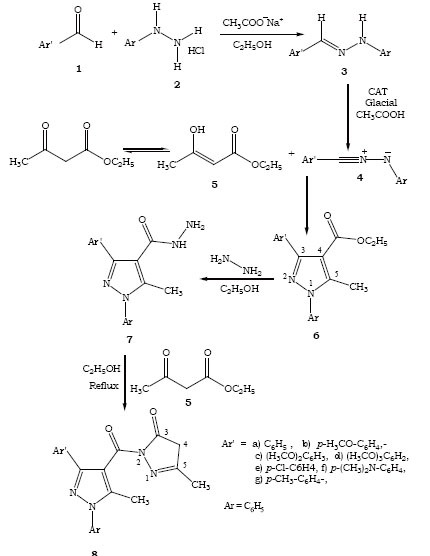
Synthesis of pyrazole derivatives.

The condensation of aromatic aldehydes 1 and phenyl hydrazine hydrochloride 2 in ethyl alcohol in the presence of sodium acetate give the corresponding aldehyde hydrazones 3. Resultant aldehyde hydrazones 3 on treatment with enolic form of ethyl acetoacetate 5 as 1,3-dipolarophile in the presence of oxidizing agent Chloramine-T (CAT) in glacial acetic acid give Ethyl 3-aryl-5-methyl-1-phenyl-1*H*-pyrazol-4-carboxylate 6. Pyrazoles 6 on refluxing with 80% hydrazine hydrate using absolute alcohol as a solvent for about 2–3 hours to produce the respective 5-methyl-1,3-diphenyl-1*H*-pyrazole-4-carboxylic acid hydrazide 7. The alcoholic solution of pyrazole acid hydrazides on heating with ethyl acetoacetate 5 to give the 5-methyl-2-(5-methyl-1,3-diphenyl-1*H*-pyrazole-4-carbonyl)-2,4-dihydro-pyrazol-3-one 8 (Figure 1).

### Bioactivity studies of synthesized pyrazole derivatives

#### Antioxidant activity

Scanning electron microscopic studies of erythrocyte oxidation. Erythrocytes were obtained from healthy donors. Heparinized blood was centrifuged at 1000 *g* for 15 min. After removal of plasma and buffy coat, the erythrocytes were washed thrice with PBS (20 mM, pH 7.4, NaCl – 0.9%) at room temperature and resuspended in PBS four times its volume for subsequent analysis (24). Erythrocytes were preincubated with samples (0.5 mg/mL) 5-methyl-2-(5-methyl-1,3-diphenyl-1*H*-pyrazole-4-carbonyl)-2,4-dihydropyrazol-3-one 8a-g which were dissolved in PBS containing 0.25% DMSO for 5 min. These concentrations of DMSO were found to have no effect on erythrocytes. Then hydrogen peroxide (30 mM), ferric chloride (80 μM) and ascorbic acid (50 μM) were added and incubated at 37°C for 1 hour. The reaction mixture was gently shaken while being incubated (25). Then the cells were fixed overnight at 4°C with glutaraldehyde in normal saline, reaching a final fixation concentration of about 2.4%. The cells were washed in saline solution and then dehydrated using ascending grades of alcohol (10–100%). Few drops of each sample were placed on A-1 glass cover slips, air dried at room temperature, gold coated and examined in a scanning electron microscope.

**DPPH radical scavenging assay.** The effect of the samples 8a-g in addition to the standard antioxidant butylated hydroxyl toluene (BHT) on DPPH radical was estimated according to the method of Lai *et. al* (26). Samples solubilized in methanol (0–50 μg/mL for samples 8a-g; 0–5 μg/mL for BHT) in 200 μL aliquot was mixed with 100 mM Tris-HCl buffer (800 μL, pH 7.4) and then added 1 mL of 500 μM DPPH in ethanol (final concentration of 250 μM). The mixture was shaken vigorously and left to stand for 20 min at room temperature in the dark. The absorbance of the resulting solution was measured spectrophotometrically at 517 nm. The capability to scavenge DPPH radical was calculated using the following Equation 1.

$$\text{DPPH}\;\text{Scavenging}\;\text{activity}\;(\%)=\frac{{\text{A}}_{\text{control}}-{\text{A}}_{\text{sample}}}{{\text{A}}_{\text{control}}}\times 100$$

***Measurement of reducing power.*** The reducing power of samples 8a-g was determined according to the method (27) of Yen and Chen. The samples 8a-g (0–50 μg/mL) were mixed with an equal volume of 0.2 M phosphate buffer, pH 6.6 and 1% potassium ferricyanide. The mixture was incubated at 50°C for 20 min. Then an equal volume of 10% trichloroacetic acid was added to the mixture and then centrifuged at 5000 g for 10 min. The upper layer of solution was mixed with distilled water and 0.1% ferric chloride at a ratio of 1:1:2 and the absorbance were measured at 700 nm. Increased absorbance of the reaction mixture indicated increased reducing power.

***DNA protection assay.*** DNA protection ability of samples 8a-g was performed using lambda phage DNA (28). Briefly, λ phage DNA (0.6 μg) was subjected to oxidation using Fenton’s reagent (0.3 mM hydrogen peroxide, 0.5 μM ascorbic acid and 0.8 μM ferric chloride) in presence and absence of the sample (0.2 mg) for 2 hours at 37°C. The samples 8a-g was subjected for electrophoresis (Submarine electrophoresis system, Bangalore Geni, Bangalore, India) on 1% agarose for 2 hours at 50 volts DC. Gels were stained with ethidium bromide (0.5 μg/mL) and documented (Herolab, Germany).

**Statistical analysis.** All the experiments were carried out in triplicates (n=3) and the results are expressed as mean ± standard deviation (SD).

#### Antimicrobial activity

**Antibacterial activity assay by paper disc diffusion method (29).** Synthesized Pyrazoles (8a-g) were screened (dose of 100 μg) for their antibacterial activity against Gram-negative bacteria *Escherichia coli* (*E. coli*) and Gram-positive bacteria (*S. aureus*) using filter paper disc method. Plates inoculated with *E. coli* were incubated for 48 hr and plates inoculated with *S. aureus* for 24 hr respectively at room temperature. Streptomycin sulphate was used as a standard. After the period of incubation the inhibition zones were measured in mm and results obtained are shown in Table 1.

**Antifungal activity assay.** All the compounds were also screened (dose of 100 μg) for their antifungal activity against *C. albicans* and *A. niger* using Griseofulvin as a standard. The results are shown in Table 1.

**Table 1:** Antibactrial and Antifungal activity of synthesized pyrazole (8a-g) derivatives (Zone of inhibition in mm)

Componds	Antibactrial activity		Antifungal activity	
	*E. coli*	*S. aureus*	*C. albicans*	*A. niger*

8a	08	10	06	06
8b	12	12	08	06
8c	10	08	08	04
8d	10	08	06	04
8e	12	10	08	06
8f	14	12	10	08
8g	12	10	06	04
Streptomycin Sulpate	18	20	Not tested	Not tested
Griseofulvin	Not tested	Not tested	14	12

## RESULTS AND DISCUSSION

**Figure 2:**
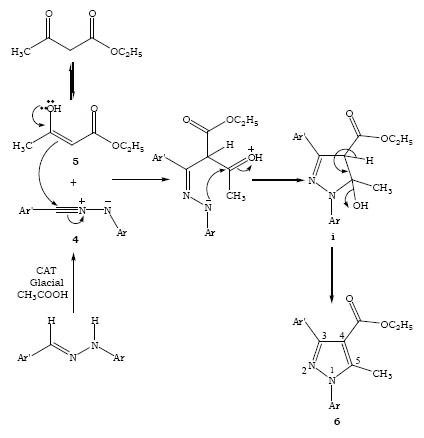
Mechanism for the formation of pyrazole.

The pyrazoles 5-methyl-2-[5-methyl-1,3-diphenyl-1*H*-pyrazole-4-carbonyl]-2,4-dihydro-pyrazol-3-one starting from enolic form of ethyl acetoacetate 5 as 1,3-dipolarophile with the cycloaddtion of *in situ* generated nitrile imine 4 by the catalytic dehydrogenation of diphenyl hydrazones 3a using CAT to get the cycloadducts Ethyl 5-methyl-1,3-diphenyl-1*H*-pyrazole-4-carboxylate 6a via the intermediate i pyrazolines, it was observed that the aromatisation is the driving force for the elimination of water molecule (Figure 2).

In typical reaction, a mixture of aldehyde hydrazone 3a (2.35 g, 12.0 mmoles), excess of ethyl acetoacetate 5 (2.6 g, 20.0 mmoles) and CAT (3.94 g, 14.0 mmoles) in glacial acetic acid and stirred at room temperature for about 2–3 hours. After the usual work up, the reaction afforded 6a as light yellow oil in 80% (2.93 g) yield. IR, ^1^H NMR, ^13^C NMR, MS studies and elemental analysis provide the structural proof for the products. For instance, in IR spectra, the peak expected due to -OH group in the region 3550–3640 cm^−1^ was found absent and it shows ester carbonyl stretching frequency at 1716–1728 cm^−1^ and a C=N frequency at 1608–1632 cm^−1^. In ^1^H NMR spectra, the signal due to -OC_2_H_5_ protons appears as a quartet in the region δ 4.12–4.31 ppm, (2H for -O-CH_2_-.group) and a triplet in the region 1.18–1.30 ppm, (3H for -OCH_2_-CH_3_), while the vinylic -CH_3_ protons appear as a singlet in the region δ 2.68–2.75 ppm. is probably due to deshielding by -CO-OC_2_H_5_ group. These observations support the formation of the cycloadducts 6 with the loss of water molecule. In ^13^C NMR spectra, the -C_3_ and -C_4_-carbon appear as singlet (decoupled) in the region δ 160.82–161.14 and δ 108.32–118.86 ppm respectively, while C_5_-carbon appear as singlet in the region δ 176.14–176.26 ppm. All cycloadducts showed MH^+^ as a base peak in the mass spectra and significantly stable molecular ion peaks with the relative abundance ranging from 20–90%, which strongly favors the formation of the cycloadducts.

The obtained Ethyl 5-methyl-1,3-diphenyl-1*H*-pyrazole-4-carboxylate 6a on refluxing with 80% hydrazine hydrate in presence of absolute alcohol for about 2–3 hours give 3-aryl-5-methyl-1-phenylpyrazole-4-carboxylic acid hydrazide 7a. The structural proof for acid hydrazide 7a confirmed by IR and ^1^H NMR spectra. In IR Spectra, the ester carbonyl stretching frequency at 1716–1728 cm^−1^ was found absent but it shows carbonyl hydrazine frequency at 1696–1708 cm^−1^ and in ^1^H NMR spectra it shows the absence of quartet in the region δ 4.12–4.31 ppm, (2H for -O-CH_2_-.group) and a triplet in the region δ 1.18–1.30 ppm, (3H for -OCH_2_-CH_3_).

**Figure 3:**
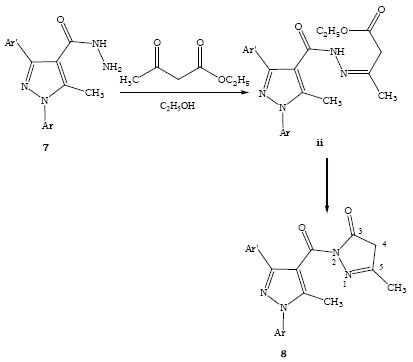
Synthesis of pyrazolone derivatives.

To extend the utility of the 3-aryl-5-methyl-1-phenylpyrazole-4-carboxylic acid hydrazide heated with ethyl acetoacetate in absolute alcohol to get the cycloadducts 5-methyl-2-(5-methyl-1,3-diphenyl-1*H*-pyrazole-4-carbonyl)-2,4-dihydro-pyrazol-3-one (8a) via uncyclized intermediate ii (Figure 3).

The formation of 5-methyl-2-(5-methyl-1,3-diphenyl-1*H*-pyrazole-4-carbonyl)-2,4-dihydropyrazol-3-one (8a-g) structures confirmed by IR, ^1^H NMR, ^13^C NMR and elemental analysis. For instance, in IR spectra, the 3-carbonyl stretching frequency shows at 1696–1710 cm^−1^ and -C=N frequency at 1588–1612. In ^1^H NMR spectra, the signal due to -CH_2_- protons appears as a singlet in the region δ 2.68–2.96 ppm, (2H for -CH_2_-.group) and methyl protons appears as a singlet in the region 0.96–1.08 ppm, (3H for -CH_3_). These observations support the formation of the cycloadducts 8. In ^13^C NMR spectra, the -C_4_ and -C_5_-carbon appear as singlet (decoupled) in the region δ 35.12–35.88 and δ 155.56–156.04 ppm respectively, while carbonyl carbon -C_3_ appear as singlet in the region δ 170.14–170.46 ppm. This strongly favors the formation of the cycloadducts 8a-g.

### Effect of samples 8a-g on Erythrocyte Oxidation

**Figure 4:**
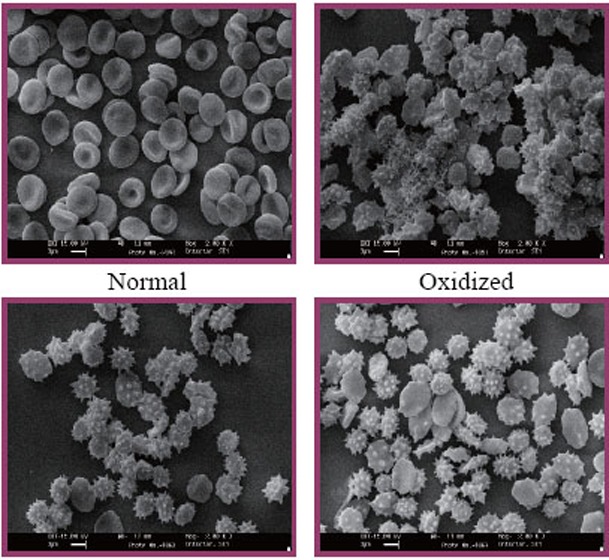
Scanning electron micrographs of erythrocyte membrane oxidation studies.

The effect of samples 8a-g was studied for their preventive effect on erythrocyte oxidation using the method as described in materials and methods. The scanning electron micrographs (Figure 4) show the protective ability of 8a and 8f samples on erythrocyte membrane oxidation. As compared to normal erythrocytes, erythrocytes treated with hydrogen peroxide showed the appearance of echinocytes and also agglutination indicating damage to the cell membrane. In 8a-g samples the presence of normal cells can also be seen in addition to oxidized cells indicating the protective role of these compounds. The protection may not be comparable to that of the normal cells, but compared to the oxidized erythrocytes protection by the tested compound is evident.

### Antioxidant activity of samples 8a-g

The antioxidant activity of samples 8a-g was evaluated by DPPH radical scavenging, reducing power and DNA protection assays. The free radical scavenging ability of samples 8a-g was evaluated by DPPH scavenging model system using the Equation 1 (Table 2). All the samples showed free radical scavenging ability, but when compared with the standard antioxidant the samples tested showed 50% lesser activity. These results indicate the potential electron donating ability of samples.

**Table 2:** DPPH Radical Scavenging activity of samples 8a-g and standard antioxidant BTH

Samples	Concentration of Sample (μg/mL)	% Radical Scavenging activity a

Control	0	0.00 ± 0.00
8a	10	5.38 ± 0.77
	20	12.61 ± 1.01
	30	18.59 ± 1.04
	40	25.98 ± 0.96
	50	31.91 ± 1.80
8b	10	6.01 ± 2.07
	20	14.43 ± 1.12
	30	21.70 ± 1.85
	40	28.93 ± 1.01
	50	35.69 ± 1.54
8c	10	5.99 ± 1.29
	20	15.52 ± 0.78
	30	21.37 ± 0.83
	40	28.06 ± 1.25
	50	34.39 ± 0.84
8d	10	5.81 ± 0.76
	20	14.26 ± 0.78
	30	20.36 ± 0.89
	40	27.55 ± 1.00
	50	33.80 ± 1.23
8e	10	5.47 ± 1.07
	20	13.16 ± 1.18
	30	19.10 ± 1.24
	40	26.57 ± 0.81
	50	32.42 ± 1.45
8f	10	6.01 ± 1.06
	20	13.48 ± 2.34
	30	23.51 ± 3.04
	40	30.35 ± 1.63
	50	37.22 ± 2.45
8g	10	5.56 ± 0.68
	20	13.84 ± 1.37
	30	19.97 ± 2.71
	40	27.04 ± 0.99
	50	33.09 ± 1.41
BTH	10	20.12 ± 0.76
	20	34.39 ± 1.16
	30	50.78 ± 2.12
	40	63.68 ± 1.28
	50	72.37 ± 1.06

a Values are expressed as mean ± standard deviation (n=3).

In addition, reducing power of samples 8a-g was also evaluated (Table 3) for their ability to reduce ferric chloride and potassium ferricyanide complex. At the initial concentrations (10–20 μg/mL) there was no significant differences in the activity were observed. However, as the concentration was increased (30–50 μg/mL) 8f showed higher reducing power and 8a showed lower reducing power. The increased absorbance at 700 nm indicated the presence of reducing power.

**Table 3:** Reducing power of samples 8a-g and standard antioxidant BHT

Samples	Concentration of Sample (μg/mL)	Absorbance at 700 nm (in optical density)^a^

8a	10	0.265 ± 0.013
	20	0.314 ± 0.014
	30	0.337 ± 0.016
	40	0.375 ± 0.013
	50	0.417 ± 0.012
8b	10	0.304 ± 0.008
	20	0.334 ± 0.008
	30	0.384 ± 0.007
	40	0.424 ± 0.006
	50	0.466 ± 0.006
8c	10	0.292 ± 0.006
	20	0.341 ± 0.005
	30	0.386 ± 0.005
	40	0.423 ± 0.005
	50	0.448 ± 0.005
8d	10	0.283 ± 0.009
	20	0.342 ± 0.005
	30	0.377 ± 0.004
	40	0.414 ± 0.007
	50	0.436 ± 0.009
8e	10	0.286 ± 0.008
	20	0.327 ± 0.008
	30	0.376 ± 0.007
	40	0.401 ± 0.006
	50	0.434 ± 0.007
8f	10	0.254 ± 0.011
	20	0.324 ± 0.018
	30	0.373 ± 0.010
	40	0.434 ± 0.014
	50	0.484 ± 0.021
8g	10	0.274 ± 0.007
	20	0.325 ± 0.010
	30	0.353 ± 0.009
	40	0.383 ± 0.009
	50	0.436 ± 0.008
BTH	10	
	20	
	30	
	40	
	50	

a Values are expressed as mean ± standard deviation (n=3).

**Figure 5:**
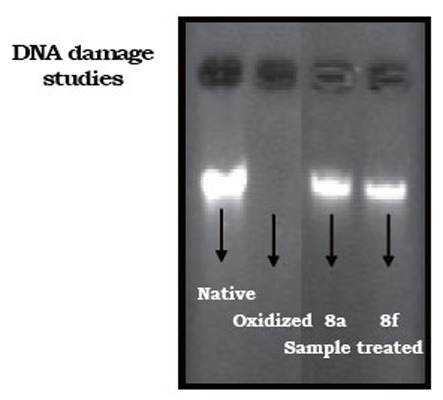
Electrophoretic mobility of DNA was different upon treatment of 8f and 8a.

Also, DNA protective ability of 8a-g were evaluated on lambda phage DNA oxidation (Figure 5). The hydroxyl radical generated by Fenton’s reagent caused DNA fragmentation with increase in its electrophoretic mobility and thereby the fragments have run out of the gel (lane 2). Upon treatment of 8f and 8a prior to oxidation the extent of DNA damage was minimized. As evidenced by gel documentation analysis, compared to native DNA (lane 1), higher protection (45–50%) was observed in 8f treated sample (lane 3), while 40–45% protection was observed for 8a (lane 4).

### Observation

It was observed that the pyrazoles (8a-g) derivatives showed less antibacterial and antifungal activity. The antibacterial activity of 8f, 8b, 8e, 8g, 8c, 8d and 8a shows Gram-negative bacteria *Escherichia coli* (*E. coli*) and Gram-positive bacteria (*S. aureus*). The antibacterial and antifungal activity of 8f is more compared to other. This indicates that, as the size and substituents increases the antibacterial and antifungal activity increases.

## EXPERIMENTAL

### Typical procedure for the preparation of Ethyl 5-methyl-1,3-diphenyl-1*H*-pyrazole-4-carboxylate (6a)

A mixture of benzaldehyde hydrazone (3 a, 2.35 g, 12.0 mmoles), excess of freshly distilled ethyl acetoacetate (5, 2.6 g, 20.0 mmoles) and CAT (3.94 g, 14.0 mmoles) in glacial acetic acid (25 ml) were stirred at room temperature for 2–3 hours. The progress of the reaction was monitored by TLC. After the completion of the reaction the residual mass was extracted into ether (25 ml), washed successively with water (2 × 20 ml), 1N NaOH (1 × 10 ml), brine solution (2 × 15 ml) and dried over anhydrous sodium sulphate. Evaporation of the solvent afforded crude oily substance, which, in TLC (chloroform : acetone : 7 :1 v/v) gave one major spot with R_f_ value 0.66, two minor spots with R_f_ values 0.58 and 0.52 corresponding to the product and starting materials respectively. Purification was done by column chromatography using benzene: ethyl acetate (8:1) as eluent, which afforded 6a as light yellow oil in 80% (2.93 g) yield. while minor as in 10% (0.36 g) yield. The pyrazole 6a showed in IR (Nujol): γ 1722 cm^−1^ (C=O), 1620 cm^−1^ (C=N), 1608 cm^−1^ (C=C); ^1^H NMR (CDCl_3_): δ 1.21 (t, 3H, -OCH_2_-CH_3_), 2.71 (s, 3H, CH_3_), 4.15 (q, 2H, -OCH_2_-CH_3_), 7.05-7.26 (s, 5H, Ar-H), 7.65-7.78 (m, 5H, Ar’-H); ^13^C NMR (CDCl_3_): δ 0.89 (q, 1C, CH_3_), 13.58 (q, 1C, -CH_2_-CH_3_), 58.72 (t, 1C, -CH_2_-), 108.64 (s, 1C, 4-C), 118.08 (d, 2C, 2″,6″-C), 122.60 (d, 2C, 3′,5′-C), 124.82 (s, 1C, 4″-C), 126.26 (d, 2C, 3″,5″-C), 128.68 (d, 2C, 2′,6′-C), 136.32 (s, 1C, 1″-C), 131.12 (d, 1C, 4′-C), 132.46 (s, 1C, 1′-C), 160.84 (s, 1C, 3-C), 176.16 (s, 1C, 5-C), 171.59 (s, 1C, CO). MS (relative intensity): m/e for C_19_H_18_N_2_O_2_; 307 (MH^+^, 100), 277(31), 233 (38), 218 (21), 194 (25), 112 (18), 103 (75), 91 (44), 88 (10), 29(28). Anal. Calcd: C, 74.50, H, 5.88, N, 9.15%. Found: C, 74.36, H, 5.72, N, 9.08%. The same procedure was used in all cases (Figure 1).

### Ethyl 3-(4-methoxyphenyl)-5-methyl-1-phenyl-1*H*-pyrazole-4-carboxylate (6b)

Obtained from 4-methoxybenzaldehyde hydrazone 1b (2.71 g, 12 mmoles), ethyl acetoacetate (2.6 g, 20.0 mmoles) as a oily substance in 78% (3.14 g) yield. IR (Nujol): γ 1716 cm^−1^ (C=O), 1608 cm^−1^ (C=N), 1596 cm^−1^ (C=C); ^1^H NMR (CDCl_3_): δ 1.18 (t, 3H, -OCH_2_-CH_3_), 2.75 (s, 3H, -CH_3_), 3.78 (s, 3H, -OCH_3_), 4.12 (q, 2H, -OCH_2_-CH_3_), 6.92 (d, 2H, Ar-H), 7.22 (d, 2H, Ar-H), 7.36-7.48 (m, 5H, Ar’-H); ^13^C NMR (CDCl_3_): δ 0.86 (q, 1C, CH_3_), 13.54 (q, 1C, -CH_2_-CH_3_), 55.80 (q, 1C, 4′-OCH_3_), 58.62 (t, 1C, -CH_2_-), 108.52 (s, 1C, 4-C), 118.18 (d, 2C, 2″,6″-C), 122.56 (d, 2C, 3′,5′-C), 124.88 (s, 1C, 4″-C), 126.22 (d, 2C, 3″,5″-C), 128.74 (d, 2C, 2′,6′-C), 136.28 (s, 1C, 1″-C), 131.08 (d, 1C, 4′-C), 132.42 (s, 1C, 1′-C), 160.82 (s, 1C, 3-C), 176.20 (s, 1C, 5-C), 171.68 (s, 1C, CO). MS (relative intensity): m/e for C_20_H_20_N_2_O_3_; 337 (MH^+^, 100), 307(32), 263 (40), 248 (20), 224 (24), 112 (16), 133 (78), 91 (46), 88 (10), 29(24). Anal. Calcd: C, 71.41, H, 5.99, N, 8.33%. Found: C, 71.38, H, 5.87, N, 8.25%.

### Ethyl 3-(3,4-dimethoxyphenyl)-5-methyl-1-phenyl-1*H*-pyrazole-4-carboxylate (6c)

Obtained from 3,4-dimethoxybenzaldehyde hydrazone 1c (2.56 g, 10 mmoles), ethyl acetoacetate (2.34 g, 18.0 mmoles) as a oily substance in 82% (2.74 g) yield. IR (Nujol): γ 1720 cm^−1^ (C=O), 1612 cm^−1^ (C=N), 1616 cm^−1^ (C=C); ^1^H NMR (CDCl_3_): δ 1.22 (t, 3H, -OCH_2_-CH_3_), 2.68 (s, 3H, -CH_3_), 3.75 (s, 6H, -OCH_3_), 4.16 (q, 2H, -OCH_2_-CH_3_), 6.98-7.12 (m, 3H, Ar-H), 7.48-7.66 (m, 5H, Ar′-H); ^13^C NMR (CDCl_3_): δ 1.02 (q, 1C, CH_3_), 13.66 (q, 1C, -CHS_2_-CH_3_), 55.76 (q, 1C, 4′-OCH_3_), 55.84 (q, 1C, 3′-OCH_3_), 59.02 (t, 1C, -CH_2_-), 108.86 (s, 1C, 4-C), 118.28 (d, 2C, 2″,6″-C), 122.52 (d, 2C, 3′,5′-C), 124.86 (s, 1C, 4″-C), 126.34 (d, 2C, 3″,5″-C), 128.78 (d, 2C, 2′,6′-C), 136.36 (s, 1C, 1″-C), 131.12 (d, 1C, 4′-C), 132.44 (s, 1C, 1′-C), 161.14 (s, 1C, 3-C), 176.24 (s, 1C, 5-C), 171.72 (s, 1C, CO). MS (relative intensity): m/e for C_21_H_22_N_2_O_4_; 367 (MH^+^, 100), 337(30), 293 (39), 278 (23), 254 (28), 163 (76), 112 (14), 91 (46), 88 (12), 29(26). Anal. Calcd: C, 68.84, H, 6.05, N, 7.65%. Found: C, 68.77, H, 5.96, N, 7.54%.

### Ethyl 5-methyl-1-phenyl-3-(3,4,5-trimethoxyphenyl)-1*H*-pyrazole-4-carboxylate (6d)

Obtained from 3,4,5-trimethoxybenzaldehyde hydrazone 1d (2.86 g, 10 mmoles), ethyl acetoacetate (2.34 g, 18.0 mmoles) as a oily substance in 81% (3.20 g) yield. IR (Nujol): γ 1718 cm^−1^ (C=O), 1618 cm^−1^ (C=N), 1618 cm^−1^ (C=C); ^1^H NMR (CDCl_3_): δ 1.26 (t, 3H, -OCH_2_-CH_3_), 2.70 (s, 3H, -CH_3_), 3.71 (s, 9H, -OCH_3_), 4.22 (q, 2H, -OCH_2_-CH_3_), 6.96 (m, 2H, Ar-H), 7.52-7.68 (m, 5H, Ar′-H); ^13^C NMR (CDCl_3_): δ 1.04 (q, 1C, CH_3_), 13.62 (q, 1C, -CH_2_-CH3), 56.6 (q, 2C, 3′,5′-OCH_3_), 58.4 (q, 1C, 4′-OCH_3_), 59.22 (t, 1C, -CH_2_-), 108.66 (s, 1C, 4-C), 122.28 (d, 2C, 2″,6″-C), 124.66 (s, 1C, 4″-C), 126.48 (d, 2C, 3″,5″-C), 128.56 (d, 2C, 2′,6′-C), 136.54 (s, 1C, 1″-C), 131.24 (d, 1C, 4′-C), 132.46 (s, 1C, 1′-C), 136.12 (d, 2C, 3′,5′-C), 161.04 (s, 1C, 3-C), 176.22 (s, 1C, 5-C), 169.88 (s, 1C, CO). MS (relative intensity): m/e for C_22_H_24_N_2_O_5_; 397 (MH^+^, 100), 367(32), 323 (42), 308 (24), 284 (22), 193 (76), 112 (21), 91 (46), 88 (14), 29(30). Anal. Calcd: C, 66.65, H, 6.10, N, 7.07%. Found: C, 66.56, H, 5.98, N, 7.04%.

### Ethyl 3-(4-chlorophenyl)-5-methyl-1-phenyl-1*H*-pyrazole-4-carboxylate (6e)

Obtained from 4-chlorobenzaldehyde hydrazone 1e (2.76 g, 12 mmoles), ethyl acetoacetate (2.6 g, 20.0 mmoles) as a oily substance in 79% (3.21 g) yield. IR (Nujol): γ 1724 cm^−1^ (C=O), 1622 cm^−1^ (C=N), 1612 cm^−1^ (C=C); ^1^H NMR (CDCl_3_): δ 1.25 (t, 3H, -OCH_2_-CH_3_), 2.74 (s, 3H, -CH_3_), 4.22 (q, 2H, -OCH_2_-CH_3_), 6.98 (d, 2H, Ar-H), 7.18 (d, 2H, Ar-H), 7.44-7.60 (m, 5H, Ar′-H); ^13^C NMR (CDCl_3_): δ 1.02 (q, 1C, CH_3_), 13.58 (q, 1C, -CH_2_-CH_3_), 59.22 (t, 1C, -CH_2_-), 108.48 (s, 1C, 4-C), 123.36 (d, 2C, 2″,6″-C), 124.36 (s, 1C, 4″-C), 127.22 (d, 2C, 3″,5″-C), 128.62 (d, 2C, 2′,6′-C), 134.62 (d, 1C, 4′-C), 132.46 (s, 1C, 1′-C), 136.12 (d, 2C, 3′,5′-C), 138.14 (s, 1C, 1″-C), 161.84 (s, 1C, 3-C), 176.14 (s, 1C, 5-C), 169.88 (s, 1C, CO). MS (relative intensity): m/e for C_19_H_17_N_2_O_2_Cl ; 341 (MH^+^, 100), 311 (30), 267 (41), 252 (18), 228 (34), 137 (76), 112 (16), 91 (42), 88 (12), 29(26). Anal. Calcd: C, 66.96, H, 5.03, N, 8.22%. Found: C, 66.91, H, 4.90, N, 8.16%.

### Ethyl 3-(4-*N,N*-dimethylphenyl)-5-methyl-1-phenyl-1*H*-pyrazole-4-carboxylate (6f)

Obtained from 4-*N,N*-dimethylbenzaldehyde hydrazone 1f (2.86 g, 12 mmoles), ethyl acetoacetate (2.6 g, 20.0 mmoles) as a oily substance in 78% (3.25 g) yield. IR (Nujol): γ 1728 cm^−1^ (C=O), 1630 cm^−1^ (C=N), 1620 cm^−1^ (C=C); ^1^H NMR (CDCl_3_): δ 1.28 (t, 3H, -OCH_2_-CH_3_), 2.68 (s, 3H, -CH_3_), 2.98 (s, 6H, -N-(CH_3_)_2_), 4.31 (q, 2H, -OCH_2_-CH_3_), 7.08 (d, 2H, Ar-H), 7.24 (d, 2H, Ar-H), 7.48-7.66 (m, 5H, Ar′-H); ^13^C NMR (CDCl_3_): δ 1.04 (q, 1C, CH_3_), 13.62 (q, 1C, -CH_2_-CH_3_), 44.36 (q, 2C, -N(CH_3_)_2_), 59.28 (t, 1C, -CH_2_-), 108.38 (s, 1C, 4-C), 123.16 (d, 2C, 2″,6″-C), 124.30 (s, 1C, 4″-C), 127.44 (d, 2C, 3″,5″-C), 128.56 (d, 2C, 2′,6′-C), 130.04 (d, 2C, 3′,5′-C), 132.46 (s, 1C, 1′-C), 134.78 (d, 1C, 4′-C), 138.24 (s, 1C, 1″-C), 161.12 (s, 1C, 3-C), 176.26 (s, 1C, 5-C), 169.36 (s, 1C, CO). MS (relative intensity): m/e for C_21_H_23_N_3_O_2_; 350 (MH^+^, 100), 320 (28), 276 (42), 261 (22), 237 (26), 146 (74), 112 (22), 91 (40), 88 (14), 29(30). Anal. Calcd: C, 72.12, H, 6.63, N, 12.03%. Found: C, 72.12, H, 6.51, N, 11.96%.

### Ethyl 5-methyl-1-phenyl-3-*p*-tolyl-1*H*-pyrazole-4-carboxylate (6g)

Obtained from 4-methylbenzaldehyde hydrazone 1g (2.10 g, 10 mmoles), ethyl acetoacetate (2.08 g, 16.0 mmoles) as a oily substance in 80% (2.56 g) yield. IR (Nujol): γ 1726 cm^−1^ (C=O), 1632 cm^−1^ (C=N), 1624 cm^−1^ (C=C); ^1^H NMR (CDCl_3_): δ 1.30 (t, 3H, -OCH_2_-CH_3_), 2.16 (s, 3H, -CH_3_), 2.72 (s, 3H, -CH_3_), 4.26 (q, 2H, -OCH_2_-CH_3_), 7.06 (d, 2H, Ar-H), 7.28 (d, 2H, Ar-H), 7.42-7.64 (m, 5H, Ar′-H); ^13^C NMR (CDCl_3_): δ 0.96 (q, 1C, CH_3_), 13.58 (q, 1C, -CH_2_-CH_3_), 21.06 (q, 3H, -CH_3_), 59.08 (t, 1C, -CH_2_-), 108.44 (s, 1C, 4-C), 124.04 (d, 2C, 2″,6″-C), 124.44 (s, 1C, 4″-C), 127.66 (d, 2C, 3″,5″-C), 128.58 (d, 2C, 2′,6′-C), 130.18 (d, 2C, 3′,5′-C), 132.52 (s, 1C, 1′-C), 134.86 (d, 1C, 4′-C), 138.32 (s, 1C, 1″-C), 161.02 (s, 1C, 3-C), 176.16 (s, 1C, 5-C), 169.22 (s, 1C, CO). MS (relative intensity): m/e for C_20_H_20_N_2_O_2_; 321 (MH^+^, 100), 291(30), 247 (40), 232 (22), 208 (26), 164 (78), 112 (18), 91 (42), 88 (12), 29(26). Anal. Calcd: C, 74.98, H, 6.26, N, 8.74%. Found: C, 74.92, H, 6.17, N, 8.66%.

### General procedure for the synthesis of 5-methyl-1,3-diphenyl-1*H*-pyrazole-4-carboxylic acid hydrazide

Ethyl 5-methyl-1,3-diphenyl-1*H*-pyrazole-4-carboxylate (6a, 2.93 g) reflux on water bath with excess of 80% hydrazine hydrate using absolute alcohol as a solvent for about 2–3 hours to produce the respective 5-methyl-1,3-diphenyl-1*H*-pyrazole-4-carboxylic acid hydrazide (7a, 2.09 g) as light yellow oil in 75% yield.

### Typical procedure for the synthesis of 5-methyl-2-(5-methyl-1,3-diphenyl-1*H*-pyrazole-4-carbonyl)-2,4-dihydro-pyrazol-3-one (8a)

The alcoholic solution of pyrazole acid hydrazides (7a, 2.09 g) on heating with freshly distilled ethyl acetoacetate (5, 1.04 g) for about 2–3 hours. The progress of the reaction was monitored by TLC. After the completion of the reaction the residual mass was extracted into ether (25 ml) and washed successively with water (2 × 20 ml) and dried over anhydrous sodium sulphate. Evaporation of the solvent afforded crude oily substance gave one major spot with R_f_ value 0.54. The purification was done by column chromatography using chloroform : acetone : (7:1) as eluent, which afforded 8a is the respective product 5-methyl-2-(5-methyl-1,3-diphenyl-1*H*-pyrazole-4-carbonyl)-2,4-dihydropyrazol-3-one (8a, 1.99 g) in 78 % yield. The obtained pyrazolone 8a showed in IR (Nujol): γ 1701 cm^−1^ (C=O) and 1592 cm^−1^ (C=N); ^1^H NMR (CDCl_3_): δ 0.98 (s, 3H, -CH_3_), 2.71 (s, 2H, --CH_2_-), 6.05-7.78 (m, 10H, Ar & Ar′-H); ^13^C NMR (CDCl_3_): δ 20.02 (q, 1C, CH_3_), 35.58 (q, 1C, 4-CH_2_-), 157.64 (s, 1C, 5-C), 170.76 (d, 1C, 3-C=O). Anal. Calcd: C, 70.36, H, 5.04, N, 15.64, O, 8.90 %. Found: C, 70.04, H, 5.02, N, 15.53, O, 8.78 %. The same procedure was used in all cases.

### 2-[3-(4-methoxy-phenyl)-5-methyl-1-phenyl-1*H*-pyrazole-4-carbonyl]-5-methyl-2,4-dihydro-pyrazol-3-one (8b)

Obtained from pyrazole acid hydrazides (7b, 3.08 g) on heating with freshly distilled ethyl acetoacetate (5, 1.0g) as a oily substance in 74% (2.71g) yield. IR (Nujol): γ 1698 cm^−1^ (C=O) and 1612 cm^−1^ (C=N); ^1^H NMR (CDCl_3_): δ 0.96 (s, 3H, -CH_3_), 2.68 (s, 2H, -CH_2_-), 6.08-7.86 (m, 9H, Ar & Ar′-H); ^13^C NMR (CDCl_3_): δ 20.02 (q, 1C, CH_3_), 35.12 (q, 1C, 4-CH_2_-), 155.94 (s, 1C, 5-C), 170.88 (d, 1C, 3-C=O). Anal. Calcd: C, 68.03, H, 5.19, N, 14.42, O, 12.32 %. Found: C, 67.88, H, 5.06, N, 14.16, O, 12.08 %.

### 2-[3-(3,4-dimethoxy-phenyl)-5-methyl-1-phenyl-1*H*-pyrazole-4-carbonyl]-5-methyl-2,4-dihydro-pyrazol-3-one (8c)

Obtained from pyrazole acid hydrazides (7c, 2.93 g) on heating with freshly distilled ethyl acetoacetate (5, 1.0 g) as a oily substance in 76% (2.64g) yield. IR (Nujol): γ 1710 cm^−1^ (C=O) and 1594 cm^−1^ (C=N); ^1^H NMR (CDCl_3_):δ 1.08 (s, 3H, -CH_3_), 2.86 (s, 2H, -CH_2_-), 6.04-7.88 (m, 8H, Ar & Ar′-H); ^13^C NMR (CDCl_3_): δ 19.76 (q, 1C, CH_3_), 35.66 (q, 1C, 4-CH_2_-), 155.72 (s, 1C, 5-C), 171.54 (s, 1C, 3-C=O). Anal. Calcd: C, 66.00, H, 5.30, N, 13.39, O, 15.28 %. Found: C, 65.78, H, 5.10, N, 13.16, O, 15.04 %.

### 5-methyl-2-[5-methyl-1-phenyl-3-(3,4,5-trimethoxy-phenyl)-1*H*-pyrazole-4-carbonyl]-2,4-dihydro-pyrazol-3-one (8d)

Obtained from pyrazole acid hydrazides (7d, 2.09 g) on heating with freshly distilled ethyl acetoacetate (5, 1.0 g) as a oily substance in 75% (1.83 g) yield. IR (Nujol): γ 17040 cm^−1^ (C=O) and 1602 cm^−1^ (C=N); ^1^H NMR (CDCl_3_): δ 1.02 (s, 3H, -CH_3_), 2.92 (s, 2H, -CH_2_-), 6.14-8.08 (m, 7H, Ar & Ar′-H); ^13^C NMR (CDCl_3_): δ 19.92 (q, 1C, CH_3_), 35.48 (q, 1C, 4-CH_2_-), 155.94 (s, 1C, 5-C), 170.86 (s, 1C, 3-C=O). Anal. Calcd: C, 64.22, H, 5.39, N, 12.46, O, 17.84 %. Found: C, 64.10, H, 5.16, N, 12.14, O, 17.66 %.

### 2-[3-(4-Chloro-phenyl)-5-methyl-1-phenyl-1*H*-pyrazole-4-carbonyl]-5-methyl-2,4-dihydro-pyrazol-3-one (8e)

Obtained from pyrazole acid hydrazides (7e, 2.91 g) on heating with freshly distilled ethyl acetoacetate (5, 1.0 g) as a oily substance in 72% (2.52 g) yield. IR (Nujol): γ 1694 cm^−1^ (C=O) and 1606 cm^−1^ (C=N); ^1^H NMR (CDCl_3_): δ 0.92 (s, 3H, -CH_3_), 2.90 (s, 2H, -CH_2_-), 6.10-7.86 (m, 9H, Ar & Ar′-H); ^13^C NMR (CDCl_3_): δ 20.06 (q, 1C, CH_3_), 35.44 (q, 1C, 4-CH_2_-), 155.82 (s, 1C, 5-C), 171.26 (d, 1C, 3-C=O). Anal. Calcd: C, 64.20, H, 4.36, Cl, 9.02, N, 14.25, O, 8.15 %. Found: C, 63.86, H, 4.04, Cl, 8.84, N, 14.08, O, 7.92 %.

### 2-[3-(4-dimethylamino-phenyl)-5-methyl-1-phenyl-1*H*-pyrazole-4-carbonyl]-5-methyl-2,4-dihydro-pyrazol-3-one (8f)

Obtained from pyrazole acid hydrazides (7f, 2.08 g) on heating with freshly distilled ethyl acetoacetate (5, 1.0 g) as a oily substance in 72% (1.79 g) yield. IR (Nujol): γ 1706 cm^−1^ (C=O) and 1594 cm^−1^ (C=N); ^1^H NMR (CDCl_3_): δ 1.06 (s, 3H, -CH_3_), 2.88 (s, 2H, -CH_2_-), 6.08-7.74 (m, 9H, Ar & Ar′-H); ^13^C NMR (CDCl_3_): δ 20.02 (q, 1C, CH_3_), 35.36 (q, 1C, 4-CH_2_-), 155.78 (s, 1C, 5-C), 171.16 (d, 1C, 3-C=O). Anal. Calcd: C, 68.78, H, 5.77, N, 17.42, O, 7.90 %. Found: C, 68.54, H, 5.52, N, 17.26, O, 7.68 %.

### 5-methyl-2-(5-methyl-1-phenyl-3-*p*-tolyl-1-phenyl-1*H*-pyrazole-4-carbonyl)-2,4-dihydro-pyrazol-3-one (8g)

Obtained from pyrazole acid hydrazides (7g, 3.12 g) on heating with freshly distilled ethyl acetoacetate (5, 1.0 g) as a oily substance in 70% (2.65 g) yield. IR (Nujol): γ 1694 cm^−1^ (C=O) and 1606 cm^−1^ (C=N); ^1^H NMR (CDCl_3_):δ 0.96 (s, 3H, -CH_3_), 2.80 (s, 2H, -CH_2_-), 6.02-7.94 (m, 9H, Ar & Ar′-H); ^13^C NMR (CDCl_3_): δ 19.96 (q, 1C, CH_3_), 35.54 (q, 1C, 4-CH_2_-), 155.96 (s, 1C, 5-C), 170.98 (d, 1C, 3-C=O). Anal. Calcd: C, 70.95, H, 5.41, N, 15.04, O, 8.59 %. Found: C, 70.48, H, 5.02, N, 14.86, O, 8.32 %.
